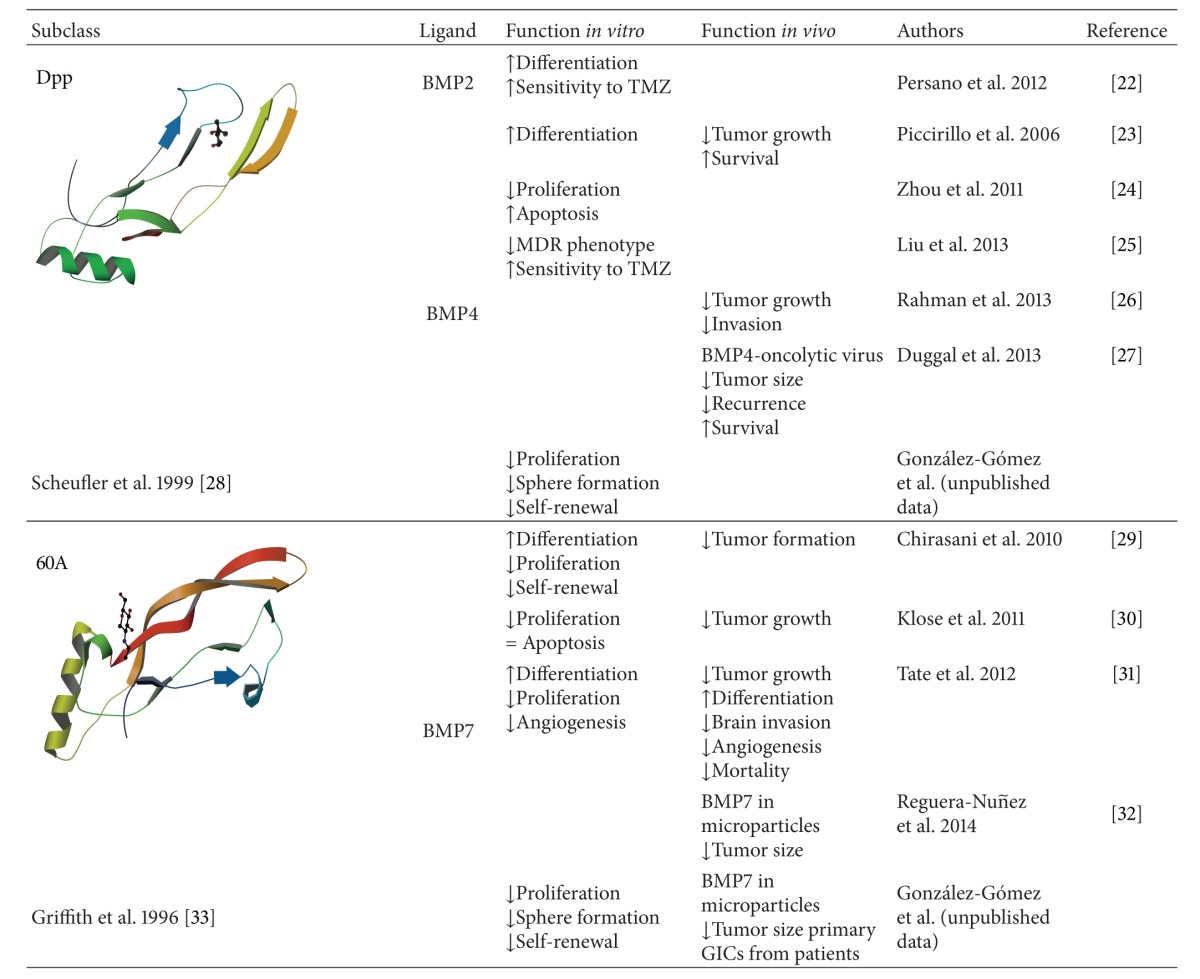# BMPs as Therapeutic Targets and Biomarkers in Astrocytic Glioma

**DOI:** 10.1155/2014/549742

**Published:** 2014-04-28

**Authors:** Pilar González-Gómez, Nilson Praia Anselmo, Helena Mira

**Affiliations:** ^1^Unidad de Neurobiología Molecular, UFIEC, Instituto de Salud Carlos III, Carretera Majadahonda-Pozuelo, Km. 2.2, Majadahonda, 28220 Madrid, Spain; ^2^Laborotório de Biologia Molecular da UFPA “Francisco Mauro Salzano”, Instituto de Ciências Biológicas, Universidade Federal do Pará, 66075-900 Belém, PA, Brazil

## Abstract

Astrocytic glioma is the most common brain tumor. The glioma initiating cell (GIC) fraction of the tumor is considered as highly chemoresistant, suggesting that GICs are responsible for glioma relapse. A potential treatment for glioma is to induce differentiation of GICs to a more benign and/or druggable cell type. Given BMPs are among the most potent inducers of GIC differentiation, they have been considered as noncytotoxic therapeutic compounds that may be of use to prevent growth and recurrence of glioma. We herein summarize advances made in the understanding of the role of BMP signaling in astrocytic glioma, with a particular emphasis on the effects exerted on GICs. We discuss the prognostic value of BMP signaling components and the implications of BMPs in the differentiation of GICs and in their sensitization to alkylating drugs and oncolytic therapy/chemotherapy. This mechanistic insight may provide new opportunities for therapeutic intervention of brain cancer.

## 1. Introduction

Astrocytic glioma is the most common tumor of the adult central nervous system. It comprises pilocytic astrocytoma (grade I), low grade astrocytoma (A, grade II), anaplastic astrocytoma (AA, grade III), and glioblastoma (GBM, grade IV) [1]. Glioblastoma is further subdivided into primary GBM, which arises de novo in older patients in the absence of a preexisting low grade lesion, and secondary GBM, which most often develops in younger adults through malignant progression from low grade A, to AA and finally to GBM [2]. Despite recent advances made in both diagnostic modalities and therapeutic strategies, astrocytic glioma remains as one of the deadliest human cancers. The 5-year survival rate in patients with this type of solid tumor is among the lowest for all cancers. The median survival for patients with GBM is about one year [3].

The major obstacle to develop more robust molecular signatures and better therapies for GBM patients arises from the high intra- and intertumor cellular heterogeneity. As in many types of solid cancers, diversity of glioma may be a consequence of genetic changes, clonal evolution, different environment, and the existence of a cellular hierarchy in which a minority of stem-like cells generate nontumorigenic more differentiated cells [4]. The Tumor Initiating Cell (TIC) model of cancer development and progression states that tumors, like normal adult tissues, contain a subset of cells characterized by three main properties: (1) self-renewal, this is, the capacity to produce more TICs, so they can maintain tumor growth indefinitely; (2) differentiation, since they give rise to differentiated progeny thereby generating all the various cell types that comprise the tumor, and (3) TICs are capable of initiating tumor growth* in vivo* [5, 6]. In the field of glial tumors, they are referred to as glioma initiating cells (GICs), and they were among the first solid tumor TICs described [7, 8]. The main hallmarks of the TIC theory are widely accepted for GICs, including their clonogenicity and capability for multilineage differentiation, activation of DNA repair mechanisms, and expression of drug transporters that might enable them to survive standard cytotoxic therapies [7, 9, 10]. Nevertheless, some studies have suggested that GICs are not a minority of the tumor cell mass and that multiple cohorts of tumor-initiating cells might be active in GBM, each one characterized by distinct functional phenotypic features and molecular profiles [11, 12]. Whatever the case, this model has generated a considerable interest because GICs appear to possess properties that make them clinically relevant. GICs have been considered as the most chemoresistant cell fraction of the tumor bulk, suggesting that they are responsible for tumor relapse.

As described above, GICs share many properties with normal neural stem cells (NSCs). Moreover GICs, like NSCs, are sensitive to developmental signaling pathways such as the Bone Morphogenetic Protein (BMP), Notch, and Wnt pathways [13]. Knowing that any therapy that fails to eliminate GICs will result in recurrence of the tumor, it is clear that new drugs that specifically target these cells are urgently needed. A potential treatment for glioma is thus to exploit developmental pathways in order to induce differentiation of GICs to a more benign phenotype that would be amenable to standard therapy. The specific focus of this review is the role of BMP proteins and their receptors in astrocytic glioma pathogenesis because: (1) some members of the BMP family of ligands have been found differentially expressed in tumors* versus* healthy tissue with a neat clinical relevance, (2) activation of the BMP pathway reduces glioma cell proliferation* in vitro* and* in vivo* and induces differentiation of the glioma initiating cells (GICs), and (3) BMPs render GICs more susceptible to conventional therapy, so BMP treatment is being considered as a promising therapeutic tool against GBM.

## 2. The BMP Signaling Pathway

Bone morphogenetic proteins are a family of proteins that were originally identified to induce bone and cartilage formation in ectopic skeletal sites* in vivo* [14, 15]. Today we know that BMPs belong to the TGFb superfamily of cytokines and that they are pleiotropic molecules that exert a variety of effects in the whole body due to the high degree of promiscuity in the interaction of ligands with their receptors and regulators (reviewed by Kim and Choe [16]). For instance, it has been demonstrated that some BMPs are implicated in the development of several cancers, sometimes being ligated to tumor progression while others playing a role as tumor suppressors [17].

BMP ligands exert their activities by way of serine-threonine kinase receptors. Prior to that they have to be cut in the cytoplasm and secreted. The receptors form a tetrameric complex composed of two type II receptors (BMPRII) and two type I receptors, the prototypic ones being BMPRIA (ALK3) and BMPRIB (ALK6) [18]. BMPRIA preferentially binds BMP ligands of the Dpp subclass (BMP2/4) whereas BMPRIB has preference for members of the 60A subclass (BMP5/6/7/8). Once a BMP ligand is bound to the receptors, BMPRII phosphorylates BMPR type I, which triggers the signaling cascade by releasing and phosphorylating R-Smads. Phospho-R-Smad1,5,8 in turn oligomerize with Smad4 to form a complex that translocates to the nucleus where it starts the transcriptional response through the activation or repression of BMP-specific target genes [18].

## 3. Expression and Clinical Significance of BMP Signaling Pathway in Astrocytic Glioma

As we summarize in Table 1, BMP proteins and BMP signaling components are arising in recent studies as novel biomarkers with important therapeutic implications for astrocytic glioma. One of the largest studies exploring the value of BMPs was performed in 2013 by Wu and Yao [20]. They evaluated the expression status of BMP4 in a total of 630 patients with glioma and correlated this dataset with clinical prognosis. By both WB and RTqPCR analysis, they showed that BMP4 expression was significantly lower in tumor tissue than in adjacent healthy tissue. Moreover, BMP4 was downregulated in high grade glioma when compared with low grade glioma. Univariate analysis showed that low BMP4 levels correlated with high expression of the cell cycle marker Ki67, as well as with high tumor grade (*P* < 0.001 for both correlations). Kaplan-Meier analysis showed that patients with high BMP4 expression had significantly better prognosis (*P* < 0.001), highlighting the relevance of BMP4 as a predictor of survival [20].

In the same year, Bao et al. [21] had access to the whole genome mRNA expression microarray data of 220 glioma samples from the Chinese Glioma Genome Atlas (CGGA) database [21]. They found that BMP4 overexpression was significantly associated with low grade tumors. The correlation was validated in previously published microarray datasets from three additional databases (The Cancer Genome Atlas, TCGA; the Repository for Molecular Brain Neoplasia Data, Rembrandt; and GSE16011). Besides, they determined the protein expression level of BMP4 in an independent cohort of 77 glioma patients by immunohistochemistry (IHC), further demonstrating that BMP4 showed a low grade glioma preference both at the mRNA and protein level. The associations of BMP4 expression with clinical-pathological factors and prognosis of glioma patients were also statistically analyzed by Bao and coworkers [21]. Kaplan-Meier survival curves from the CGGA database data and also from the other 3 datasets indicated that high BMP4 expression was significantly associated with lower mortality, particularly when analyzing high grade tumors (AA and GBM). They also found a preferential expression of BMP4 in patients with isocitrate dehydrogenase 1 (IDH1) gene mutation [34], as well as in patients with a molecular signature corresponding to a proneural GBM subtype or G1 subtype, all of them features of a better prognostic GBM [35–37].

In 2009, Liu et al. reported that an active BMP signaling pathway could be beneficial for the outcome of GBM patients [19]. Using Smad1,5,8 phosphorylation as a readout, they reported that BMP signaling was significantly decreased in AA and GBM samples when compared with normal brain and low grade astrocytomas. The expression of the BMPRIB receptor was also downregulated in high grade gliomas. Moreover, Kaplan-Meier survival curves and log-rank analysis showed that patients with a low P-Smad1,5,8/total Smad1,5,8 ratio had statistically shorter survival times, reinforcing the negative correlation between P-Smad/BMPRIB and the malignant grade of glioma. In line with this finding, Lee et al. [38] demonstrated that astroglial differentiation of GIC-like cell lines is impaired in a subset of GBMs due to the epigenetic silencing of BMPRIB, strengthening the view that loss of BMP signaling contributes to the pathogenesis of glioma.

But not all reports associate strong BMP signaling or BMP levels with a better clinical outcome of glioma patients. In 2009, Liu and coworkers reported that BMP2 expression became significantly higher as the glioma's grade advanced (*P* < 0.001) and the Karnofsky Performance Status (KPS) score decreased [39]. Univariate analyses of each factor with the Cox log-rank showed that the median survival of patients with a high BMP2 expression level was significantly shorter than those with a low BMP2 expression level (*P* < 0.0001). Liu et al. claimed that BMP2 was not only a significant predictor of survival in high grade gliomas but also in lower grade gliomas. Although these authors concluded that BMP2 is a highly sensitive biomarker for glioma prognosis, this work was done in a relatively small cohort of 98 glioma patients that were all classified as primary glioma cases, since the onset of the disease was less than three months before diagnosis and there was no prior history of malignant astrocytoma. Additional studies employing larger microarray databases available nowadays should confirm the view that the role of BMP4 and BMP7 in glioma differs from that of BMP2. Future studies may also address whether or not BMP function differs between primary and secondary gliomas.

## 4. BMP Effects on Glioma Initiating Cells

The discovery of GICs and GIC regulation has been fundamental to our current understanding of glioma recurrence. A number of pathways that are commonly deregulated in glioma, including the BMP pathway, are also involved in differentiation of normal NSCs, raising the idea that it is possible to force GICs to differentiate upon restoration of or exposure to the appropriate signals.

In the developing central nervous system, BMP signaling is critical for progenitor cell specification and maintenance of a particular phenotype through dynamic transcriptional regulation [40]. In NSCs derived from early embryos, BMPs appear to promote proliferation and neuronal differentiation. In contrast, NSCs derived from older animals undergo either astrocytic differentiation or quiescence in response to BMPs [41–43]. The same regulatory networks may be important for GICs.

In a seminal study by the Vescovi group, BMPs were shown to block proliferation and promote differentiation of NSCs and GICs, thereby inhibiting tumor growth [23]. Amongst all the BMP ligands tested, BMP4 elicited the strongest effect. BMP4 effectively reduced the* in vitro* proliferation of CD133+ cells (a marker frequently used to isolate GICs) without affecting apoptosis. Accordingly, results from our group employing five different primary GBM cultures indicate that BMP4 inhibits both GIC proliferation and self-renewal (González-Gómez and Mira, unpublished data). Most importantly, the Vescovi group also demonstrated that* in vivo* delivery of BMP4 inhibits tumor growth. Mice intracranially injected with untreated glioma cells died after three to four months, but nearly all mice injected with BMP4-treated cells survived until the end of the experiment [23].

Zhou et al. [24] observed that BMP4 may act as a key inhibitory regulator of cancer initiation and therefore may be used in combined stem cell-based therapy as a noncytotoxic therapeutic agent. The CD133+ GIC fraction used in this study was isolated from the human glioma cell line U87 by using vincristine and was exposed to the BMP4 protein. They showed that BMP4 inhibited U87 GIC proliferation (*P* < 0.01) via downregulation of cyclin D1 level and promoted GIC apoptosis through induction of Bax expression and inhibition of Bcl-2 and Bcl-xL levels.

BMP4 signaling in GICs may be enhanced by means of the inhibition of metabotropic glutamate receptors (mGluRs). These receptors are predominantly involved in maintaining cellular homeostasis in the central nervous system, but evidences suggesting other functional roles in human malignancies have pointed to mGluRs as novel cancer targets [44]. Purified GICs express mGlu3 receptor, the activation of which restrains the prodifferentiating activity of BMP4* via* a mechanism of receptor-receptor interaction. Systemic treatment with an mGlu2/3 receptor antagonist reduces the growth of brain tumors originating from U87MG glioma cells [45] or human GICs in mice [46]. The antagonist limits the growth of GIC xenografts and promotes astroglial differentiation mediated by BMP4 [46], suggesting that inhibition of mGluRs may be exploited as a tool to enhance BMP signaling/GIC differentiation. More recent findings suggest that mGlu3 receptor antagonists act synergistically with DNA-alkylating agents (Temozolomide) in killing GICs [47]. Together these data highlight a novel crosstalk between mGluR3 and BMP4 and suggest that mGlu3 receptor blockade may be combined with BMP delivery as a strategy for the treatment of malignant gliomas. This is an attractive approach that warrants further investigation [44].

Chirasani et al. [29] clearly demonstrated* in vivo* and* in vitro* that BMP7, another member of the bone morphogenetic protein family, may be therapeutically useful by the same criteria used for BMP4. These authors showed that BMP7 (released by neural precursor cells) stimulates a canonical BMP response in stem-like glioblastoma cells. This interfered with all the key functions of GICs, reducing their ability to maintain a cellular hierarchy (the markers of undifferentiated cells CD133, Nestin, and Olig2 were lost, whereas the differentiation marker GFAP was induced), their self-renewal capacity (attenuated ability to form spheres), and their potential for tumor-initiation* in vivo*.

Klose et al. [30] focused on analyzing the effects of BMP7 during glioma cell proliferation* in vitro* and* in vivo*. In a glioma cell line (Gli36ΔEGFR-LITG) that overexpresses EGFR, they observed that BMP7 treatment decreased proliferation up to 50% through cell cycle arrest in the G1 phase but not by induction of apoptosis. This effect was mediated by the modulation of the expression and phosphorylation of cyclin-dependent kinase 2, cyclin-dependent kinase inhibitor p21, and downstream retinoblastoma protein. Furthermore,* in vivo* optical imaging of luciferase activity of Gli36ΔEGFR-LITG cells implanted intracranially into nude mice in the presence or absence of BMP7 treatment corroborated the antiproliferative effects of this cytokine. This report clearly underlines the tumor-suppressive role of BMP7 in glioma-derived cells.

Moreover, Tate et al. [31] demonstrated that a BMP7 variant (BMP7v) inhibits GBM growth* in vitro* and* in vivo*.* In vitro*, this BMP7v decreased primary human GIC proliferation, angiogenesis, and stem cell marker expression while enhancing neuronal and astrocyte differentiation marker expression. In subcutaneous and orthotopic GIC xenografts, which closely reproduce the human disease, BMP7v decreased tumor growth and stem cell markers, while enhancing astrocyte and neuronal differentiation compared with control mice. In addition, BMP7v reduced brain invasion, angiogenesis, and the associated mortality in an orthotopic glioma model.

Taken together, these results suggest that BMP4/7 may be explored as potential therapeutic agents for glioma (Table 2). However, this therapeutic approach must be viewed with caution given BMPs are mitogenic in a subset of tumors with repressed BMPRIB expression. Lee et al. [38] reported that 20% of GBM tumors display epigenetic silencing of BMPRIB due to CpG methylation in its promoter regions. In these primary human GBMs, GICs resemble a very early embryonic NSC that is apparently blocked from further stem cell development and differentiation due to an aberration in the BMP signaling pathway. As in NSCs from early developmental stages, BMP treatment of these GICs increases proliferation. Conversely, forced expression of the methylated-promoter-repressed BMPRIB restores the normal differentiation capacity of the GICs, halting proliferation and inducing their terminal differentiation. Thus, Lee and coworkers provide an example of a temporally deregulated and aberrantly fixed stem-like cell, with a developmental differentiation blockade, that is contributing to the pathogenesis of glioma. These observations therefore identify BMPRIB as a promising molecular therapeutic target in a subset of GBMs. The recovery of BMPRIB expression in GBM cells and the development of BMPRIB specific agonists are worthy of further investigation [38].

## 5. BMPs as Therapeutic Targets in Astrocytic Glioma

**Figure 1:**
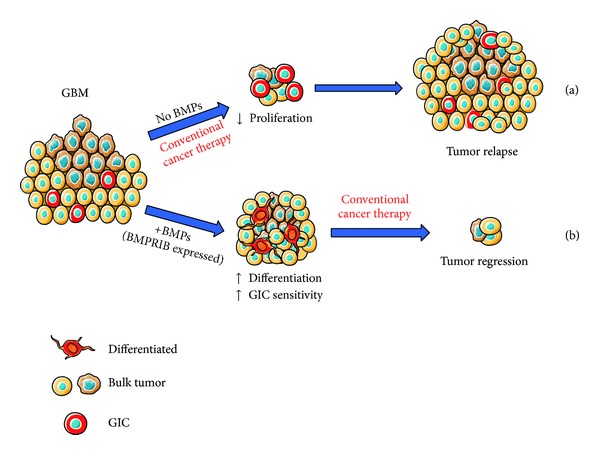
(a) Glioma initiating cells seem to be radioresistant and chemoresistant to conventional therapies and, eventually, this results in tumor recurrence. (b) One approach to target GICs in GBM could be to develop a specific chemotherapeutic agent (such as BMPs or newly synthesized molecules mimicking BMPs) able to induce GICs to differentiate into cells more amenable to standard therapy. The expression of BMPRIB would be key for inducing differentiation of GICs.

With the advent of molecular biology and the consequent improved understanding of basic tumor biology, targeted therapies have become cornerstones for cancer treatment. As we explained above, BMPs have been shown to promote GIC differentiation and to reduce GBM proliferation* in vitro* and* in vivo* [23, 24, 29–31, 38], so they are becoming promising therapeutic tools that could be used in combination with other conventional treatments (Figure 1). This has been recently explored by several groups.

Persano and coworkers [22] found that BMP2 was not only an effective prodifferentiation treatment for GBM-derived stem cells but also that the BMP2-mediated differentiation made the tumor cells more sensitive to Temozolomide (TMZ) treatment. In fact, BMP2 or TMZ delivered separately did not promote GBM apoptosis, but both treatments together exerted a synergistic effect, causing a dramatic increase in cell death. This occurred because BMP2 decreased hypoxia-inducible factor 1 alpha (HIF1*α*) stability and consequently downregulated O-6-methylguanine-DNA methyltransferase (MGMT), a HIF1*α* target, thereby allowing the TMZ alkylating action [22].

Liu et al. [25] reported that BMP4 could reverse the multidrug resistance (MDR) phenotype of tumor cells. They generated a TMZ resistant U251 glioma cell line and observed a reduction of the BMP4 protein levels. Treating the cells with BMP4 abolished the MDR phenotype, sensitizing the cells again to TMZ and other treatments. They also corroborated this finding* in vivo*. Resistant cells were transfected with GFP-BMP4 and injected into nude mice brain. The treatment with TMZ was effective only in the mice overexpressing BMP4 [25].

BMP4 treatment has been combined with bevacizumab in GBM mouse models [26]. Bevacizumab is a humanized monoclonal antiangiogenic antibody against vascular endothelial growth factor A (VEGF-A). Although bevacizumab treatment results in a significant reduction of the tumor size and a temporary patient benefit, the prolonged antiangiogenic treatment generates progressive hypoxia, which promotes tumor resistance, increased invasion, and finally tumor recurrence [48]. In fact, two recent studies showed that bevacizumab does not increase the overall survival of GBM patients although there is an improvement on the progression-free survival times [49, 50]. Novel strategies designed to overcome the proinvasive effects of bevacizumab may still be useful, since antiangiogenic therapies not only diminish tumor size but also improve blood flow, which is important for oxygen and drug delivery [51]. Rahman et al. [26] implanted human GBM cells in the striatum of immunocompromised mice and treated them with bevacizumab and BMP4 to test whether BMP4 could prevent diffuse tumor infiltration induced by bevacizumab in a malignant glioma xenograft model. It was possibly not the best model to assess the aim, because bevacizumab treatment did not result in diffuse infiltration of human GBM in the mouse brain parenchyma. Nevertheless they observed that BMP4 did have a favorable effect on GBM: it reduced tumor size and tumor invasion although there was no synergistic effect with bevacizumab treatment [26].

Effective treatment with BMPs or any other chemotherapeutic agent is limited due to the presence of the blood-brain barrier (BBB) that tightly regulates the diffusion of endogenous molecules but also of xenobiotics. Tumor-targeted drug delivery is one of the major areas in cancer research and viruses and biomaterials have already been used to deliver BMPs with good results. Duggal and colleagues developed an oncolytic vaccinia virus that overexpressed BMP4 and tested its activity* in vitro* and in an orthotopic xenograft model of GBM. The virus overexpressing BMP4 promoted cell differentiation of primary GIC cultures derived from tumor biopsies. Interestingly, GIC differentiation further increased the replication capacity of the oncolytic virus. intracranial inoculation of the BMP4-virus at the same coordinates as the tumor cells (implanted two weeks earlier) resulted in a rapid tumor regression and improved survival of the mice. This efficacy was also confirmed in a higher tumor burden setting, when the virus was inoculated 7 weeks after tumor cell implantation [27].

Early this year, the group of García-Fuentes reported the design of an implantable microparticles system optimized for the controlled release of BMP7 as a bioinspired device against GICs. The delivery system was based on the formation of heparin-BMP7 microparticles, further entrapped in a biodegradable polyester matrix. The obtained microparticles efficiently encapsulated BMP7 and released it in a controlled manner with minimum burst effect for over two months while maintaining protein bioactivity. Released BMP7 showed a remarkable capacity to stop tumor formation in an* in vitro* GIC model [32] and strongly limited growth of GIC orthotopic xenografts in immunocompromised mice (González-Gómez and collaborators, unpublished).

In summary, the combination of conventional surgery, chemotherapy, and radiotherapy with stem cell-orientated therapy may provide a new promising treatment for reducing GBM recurrence and improving patient survival. Targeting GICs with BMPs may be an innovative way to achieve this goal. Given BMPs markedly inhibit the cancer stem-like cells in other neoplasms, both from the central nervous system such as oligodendrogliomas [52] or from nonneural origin such as prostate [53] or breast tumors [54], the development of BMP-based treatments may provide new opportunities for therapeutic intervention of different cancer types besides GBM.

## 6. Concluding Remarks

In the last 10 years, for the vast majority of cancers, tumor prognosis and response to therapy have been improved by technological advances in molecular biology. Nevertheless, astrocytic glioma patients still face a poor prognosis, with even the more advanced treatments offering very limited results. In glioblastoma, most patients undergo recurrence, possibly due to the failure to eradicate GICs. Targeting GICs has opened the door to the development of new potential clinical therapies and interventions. Given BMPs block proliferation and drive differentiation of GICs* in vitro* and in mouse models of glioma, they have been proposed as promising tumor-suppressive drugs. Delivery or expression of BMP ligands causes sustained tumor regression and greatly enhances survival in xenograft mouse models. Moreover, BMPs increase GIC responsiveness to chemotherapy through downregulation of MGMT and low BMP levels are prognostic for poor survival in human glioma. Thus, BMPs or newly synthesized molecules mimicking BMP binding to its receptors may be exploited as innovative GIC-orientated treatments for astrocytic glioma.

## Figures and Tables

**Table 1:** Clinical significance of BMP signaling pathway.

Molecule	Expression data in high grade gliomas	No patients	Methods	Clinical significance	Authors	Reference
BMP2	UP	98	IHQ	↓BMP2: ↑survival time (tumor grade independent)	Liu et al. 2009	[19]

BMP4	DOWN	630	RTqPCR/WB/IHQ	↓BMP4: ↓survival time (all grades together)	Wu and Yao 2013	[20]
	DOWN	220/77	Microarray/IHQ	↓BMP4: ↓survival time (grades III and IV)	Bao et al. 2013	[21]

BMPRIB	DOWN	64	WB	n.d	Liu et al. 2009	[19]

P-Smad 1,5,8	DOWN	64	WB	↓p-Smad: ↓survival time (tumor grade independent)	Liu et al. 2009	[19]

*Bao et al. [21] validated the data using Rembrandt database and GSE16011 microarray data. N.d.: not determined.

**Table 2:** BMP effects on glioma initiating cells.